# Assessing histone demethylase inhibitors in cells: lessons learned

**DOI:** 10.1186/s13072-017-0116-6

**Published:** 2017-03-01

**Authors:** Stephanie B. Hatch, Clarence Yapp, Raquel C. Montenegro, Pavel Savitsky, Vicki Gamble, Anthony Tumber, Gian Filippo Ruda, Vassilios Bavetsias, Oleg Fedorov, Butrus Atrash, Florence Raynaud, Rachel Lanigan, LeAnne Carmichael, Kathy Tomlin, Rosemary Burke, Susan M. Westaway, Jack A. Brown, Rab K. Prinjha, Elisabeth D. Martinez, Udo Oppermann, Christopher J. Schofield, Chas Bountra, Akane Kawamura, Julian Blagg, Paul E. Brennan, Olivia Rossanese, Susanne Müller

**Affiliations:** 1 Nuffield Department of Clinical Medicine, Structural Genomics Consortium, University of Oxford, Old Road Campus Research Building, Roosevelt Drive, Oxford, OX3 7DQ UK; 2 Nuffield Department of Medicine, Target Discovery Institute, University of Oxford, Roosevelt Drive, Oxford, OX3 7FZ UK; 3 Medical Faculty, Research and Drug Development Center, Federal University of Ceará, Rua Cel. Nunes de Melo n.1000—Rodolfo Teófilo, 60, Fortaleza, CE 430-270 Brazil; 4 Cancer Research UK Cancer Therapeutics Unit, The Institute of Cancer Research, 15 Cotswold Road, London, SM2 5NG UK; 5 Epigenetics Discovery Performance Unit, Medicines Research Centre, GlaxoSmithKline R&D, Stevenage, SG1 2NY UK; 6 Hamon Center for Therapeutic Oncology Research, and Department of Pharmacology, UT Southwestern Medical Center at Dallas, Dallas, TX 75390 USA; 7 Nuffield Department of Orthopedics, Rheumatology and Musculoskeletal Sciences, Botnar Research Centre, NIHR Oxford Biomedical Research Unit, University of Oxford, Oxford, OX3 7LD UK; 8 Chemistry Research Laboratory, 12 Mansfield Road, Oxford, OX1 3TA UK; 9 Division of Cardiovascular Medicine, Radcliffe Department of Medicine, Wellcome Trust Centre for Human Genetics, Roosevelt Drive, Oxford, OX3 7BN UK; 10 Buchmann Institute for Molecular Life Science, Goethe University Frankfurt, Riedberg Campus, Max-von-Laue-Straße 15, 60438 Frankfurt am Main, Germany

**Keywords:** Histone lysine demethylase, Chromatin, Immunofluorescence, Cell proliferation, Apoptosis, Toxicity, Epigenetics, 2-Oxoglutarate oxygenases

## Abstract

**Background:**

Histone lysine demethylases (KDMs) are of interest as drug targets due to their regulatory roles in chromatin organization and their tight associations with diseases including cancer and mental disorders. The first KDM inhibitors for KDM1 have entered clinical trials, and efforts are ongoing to develop potent, selective and cell-active ‘probe’ molecules for this target class. Robust cellular assays to assess the specific engagement of KDM inhibitors in cells as well as their cellular selectivity are a prerequisite for the development of high-quality inhibitors. Here we describe the use of a high-content cellular immunofluorescence assay as a method for demonstrating target engagement in cells.

**Results:**

A panel of assays for the Jumonji C subfamily of KDMs was developed to encompass all major branches of the JmjC phylogenetic tree. These assays compare compound activity against wild-type KDM proteins to a catalytically inactive version of the KDM, in which residues involved in the active-site iron coordination are mutated to inactivate the enzyme activity. These mutants are critical for assessing the specific effect of KDM inhibitors and for revealing indirect effects on histone methylation status. The reported assays make use of ectopically expressed demethylases, and we demonstrate their use to profile several recently identified classes of KDM inhibitors and their structurally matched inactive controls. The generated data correlate well with assay results assessing endogenous KDM inhibition and confirm the selectivity observed in biochemical assays with isolated enzymes. We find that both cellular permeability and competition with 2-oxoglutarate affect the translation of biochemical activity to cellular inhibition.

**Conclusions:**

High-content-based immunofluorescence assays have been established for eight KDM members of the 2-oxoglutarate-dependent oxygenases covering all major branches of the JmjC-KDM phylogenetic tree. The usage of both full-length, wild-type and catalytically inactive mutant ectopically expressed protein, as well as structure-matched inactive control compounds, allowed for detection of nonspecific effects causing changes in histone methylation as a result of compound toxicity. The developed assays offer a histone lysine demethylase family-wide tool for assessing KDM inhibitors for cell activity and on-target efficacy. In addition, the presented data may inform further studies to assess the cell-based activity of histone lysine methylation inhibitors.

**Electronic supplementary material:**

The online version of this article (doi:10.1186/s13072-017-0116-6) contains supplementary material, which is available to authorized users.

## Background

Histones are highly conserved proteins that play important roles, not only in compacting DNA in the cell, but which also have dynamic functions in many physiological and molecular processes. Covalent modifications of the histone tail, in particular on lysine residues, are an essential part of epigenetic mechanisms to regulate processes like DNA repair and gene transcription [1, 2]. The complex code of histone tail modifications is tightly regulated and includes a variety of modifications, most prominently acetylation and methylation of lysine and arginine residues. Enzymes that modify or bind to these residues have been implicated in a variety of diseases including cancer, inflammatory diseases, heart disease and neurodegenerative diseases [3–5]. Methylation of histone lysines was once thought by many to be irreversible, but two classes of histone lysine demethylases (KDMs) have now been identified that catalyse the removal of these methylation marks. Based on their enzymatic mechanism, the flavin-dependent demethylases of the KDM1 subfamily (lysine-specific demethylases or LSDs) are distinguished from the Jumonji C (JmjC) family of KDMs, comprising the KDM2 to KDM7 subfamilies, which belong to the superfamily of Fe(II)- and 2-oxoglutarate (2-OG)-dependent oxygenases. Demethylation of different marks has been described thereby influencing the ratios of mono-, di- and tri-methylation on H3K4, H3K9, H3K27, H3K36 and H4K20, resulting in differential effects on gene transcription. For example, H3K4me2/3 and H3K36me3 are preferentially associated with transcriptionally active genes, while H3K9me2/3, H3K27me2/3 and H4K20me3 correlate with transcriptional repression [6–8].

There is growing interest in exploring the biology and disease relevance of the KDMs with the help of well-characterized ‘probe molecules’ that show potent activity in biochemical and cellular assays as well as selectivity over other KDMs [9]. Several inhibitors for the KDM1 family have been described, with the most advanced compounds entering clinical trials [6]. A number of inhibitors have also recently been discovered for the JmjC family of demethylases, e.g. [6, 10–17]. In order to draw conclusions regarding the biological activity of new inhibitors, it must first be confirmed that their observed cellular effect is due to the inhibition of the respective enzyme rather than uncharacterized off-target activity or nonspecific effects. We have developed and evaluated an immunofluorescence-based high-content imaging assay to assess the on-target effect of demethylase inhibitors and describe here the lessons learned.

## Results

**Fig. 1:**
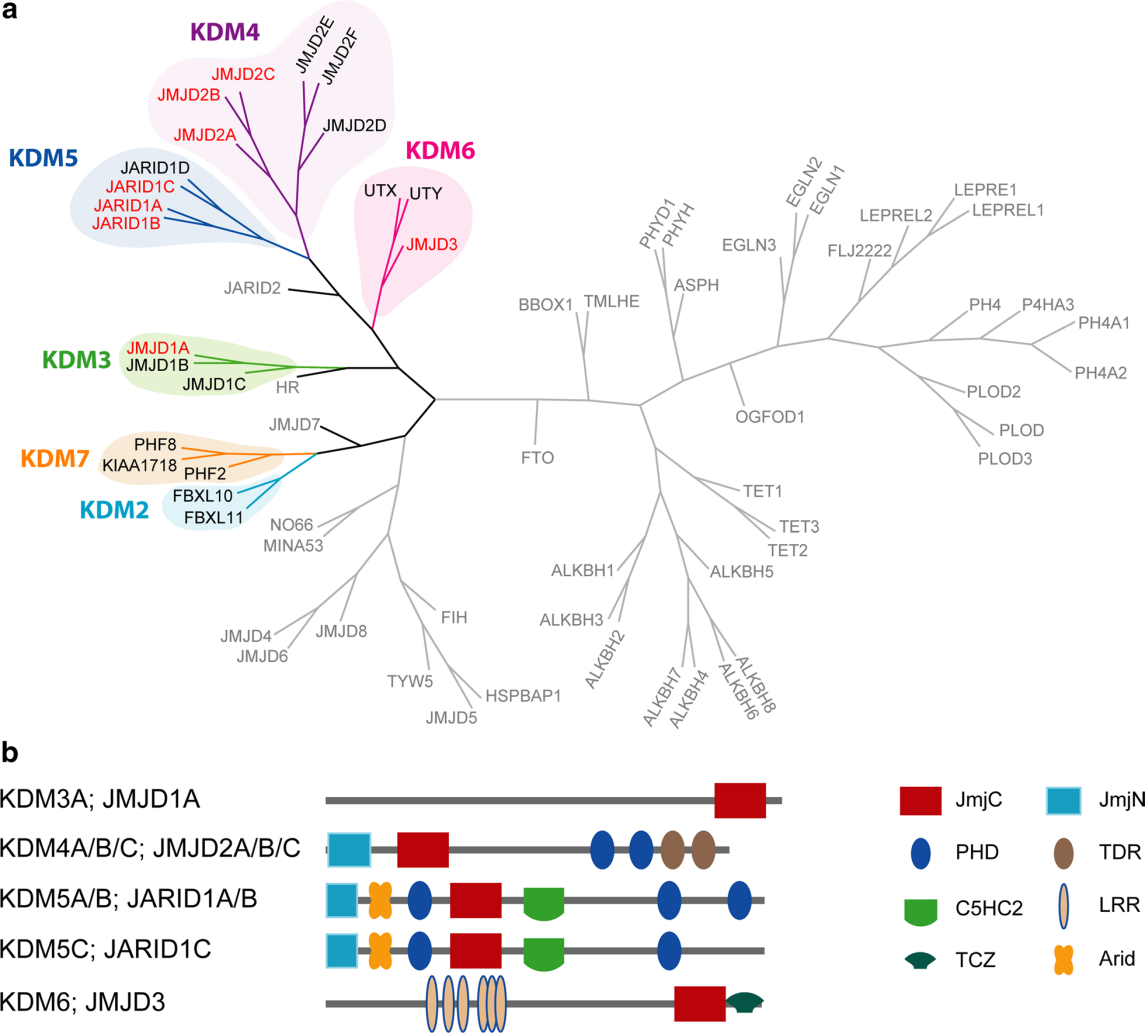
Position of KDMs in phylogenetic tree of 2-OG-dependent oxygenases. **a** Phylogenetic tree of the catalytic domains of human 2-OG oxygenases adapted according to [5]. The different subfamilies of KDMs are indicated. Non-KDM oxygenases are in *grey*. Proteins for which IF assays are described herein are highlighted in *red*. **b** Domain organization of 2-OG-dependent oxygenases for which IF assays are presented. The identities of the different domains are: *JmjC* Jumonji C domain, *JmjN* Jumonji N domain, *PHD* plant homeodomain, *TDR* tudor domain, *C5HC2* zinc finger C5HC2 type, *LRR* leucine-rich repeat, *TCZ* treble-clef zinc finger domain

Several robust in vitro biochemical assays have been developed to assess the potency of potential KDM inhibitors; however, most of them do not use the full-length protein, but rather employ truncated constructs encompassing mainly the catalytic domain. It has recently been shown that, in addition to the catalytic domain, reader domains, such as plant homeodomains (PHDs), are also important in overall KDM enzymatic activity [18, 19]. In order to assess the on-target effect of KDM inhibitors of the JmjC family in cells, we have developed a panel of high-content immunofluorescence-based assays covering the major branches of the phylogenetic tree of the JmjC family of 2-OG-dependent oxygenases that rely on the full-length protein (Fig. 1) [20]. The assays utilize an overexpression system after transiently transfecting the full-length FLAG-tagged demethylase (WT) into cells and assaying the cells overexpressing the demethylase for the respective substrate mark in a high-content immunofluorescence (IF) assay. Overexpression was found to be optimal after 20–24 h for the tested demethylases after which cell death was observed, even in the absence of inhibitor (Yapp et al., unpublished results). As control, catalytically inactive KDMs (MUT) were used, in which the specific residues involved in iron coordination were substituted for alanine residues (or tyrosine for KDM3A) (Additional file 1: Figure S1; Additional file 2: Table S1). In the ideal case, the dose–response curve of the ectopically produced WT KDM should meet the curve of the MUT KDM when the enzyme is fully inhibited by the inhibitor.

**Fig. 2:**
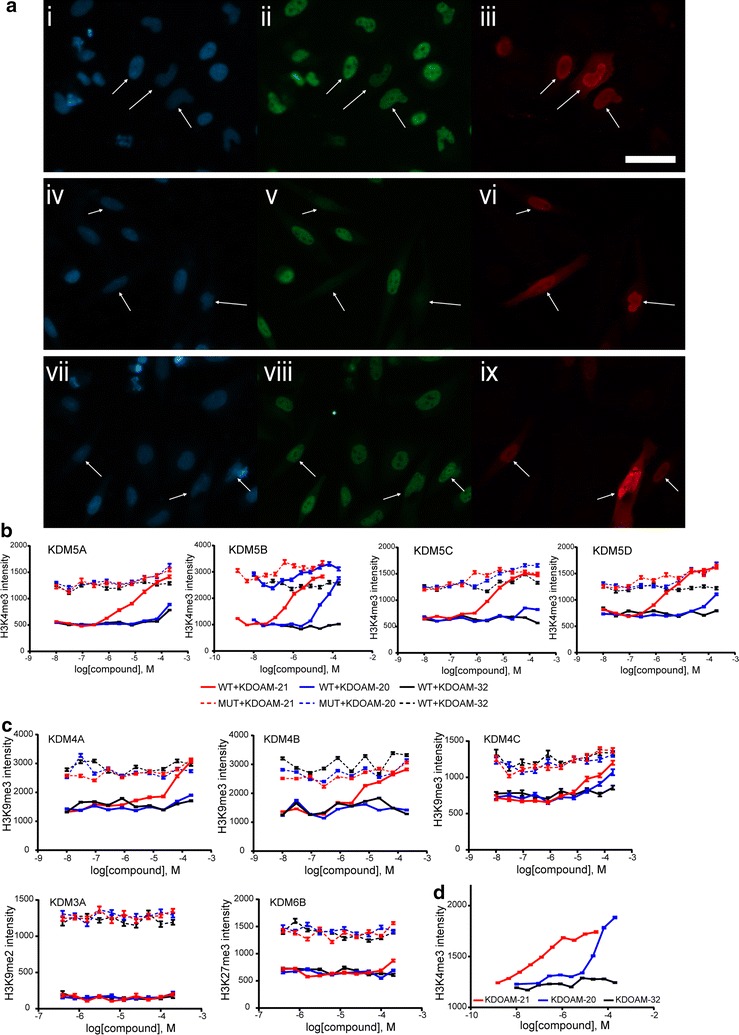
Immunofluorescence assay assessing and comparing potencies of inhibitors in cells. **a** Widefield fluorescence imaging of HeLa cells after dosing with inhibitor, fixing and staining with *i*, *iv*, *vii* DAPI (*blue*), *ii*, *v*, *viii* histone antibody for H3K4me3 (*green*), and *iii*, *vi*, *ix* a FLAG-tag antibody that demarcates cells overexpressing KDM5B (*red*). Cells overexpressing the wild-type (WT) KDM were treated with the inhibitor KDOAM-21 (*i*–*iii*) or with DMSO (*iv*–*vi*); controls cells overexpressing an inactive mutant (MUT) were treated with DMSO (*vii*–*ix*). *Arrowheads* indicate KDM overexpressing cells. The *scalebar* represents 50 µm, **b–d** measurement of the average histone mark intensity in the transfected HeLa cells allows quantification of inhibitor potency against each target. KDOAM-21 (*red*), KDOAM-20 (*blue*) and the inactive control KDOAM-32 (*black*) were tested on multiple targets, **b** illustrates the characteristic dose–response curves of the potent KDOAM-21, weak KDOAM-20 and inactive KDOAM-32 compounds on KDM5 family members. The wild type (WT) and catalytically inactive mutant (MUT) of each target are shown as *bold* and *dashed lines*, respectively. The baseline for the mutant is of similar intensity as the nontransfected cells and approximately represents complete inhibition of the target demethylase activity, **c** selectivity of KDOAM-20 and KDOAM-21 against KDM5 is further demonstrated in cells by their weaker potency against other demethylases, i.e. members of the KDM3, KDM4 and KDM6 families. In **d**, KDOAM-21 and, to a lesser extent, KDOAM-20 are shown to have activity against endogenous KDMs as seen by changes in H3K4me3 in nontransfected cells. KDOAM-32 retains its inactivity

A global decrease in methylation was observed for HeLa cervical carcinoma cells overexpressing the WT demethylase as determined by reduction in the levels of methyl-lysine antibody staining (e.g. KDM5B overexpression correlating with H3K4me3 nuclear staining in Fig. 2a iv–vi), relative to cells overexpressing the corresponding catalytically inactive MUT demethylase or nontransfected cells (Fig. 2a vii–ix).

Many reported inhibitors for the JmjC-KDMs display poor cellular activity [6, 11, 13, 14], and prodrug strategies have been utilized in an attempt to enhance cellular permeability and activity [6, 21–23]. We synthesized and tested various inhibitors from our KDM inhibitor programme as well as inhibitors described in the literature (Additional file 3: Figure S2). Recently, potent inhibitors of the KDM5 family with good cellular activity have been described by Epitherapeutics (Table 1; Additional file 3: Figure S2; Additional file 4: Table S2) [24–26]. We tested two of the reported inhibitors: KDOAM-20 and KDOAM-21 (also known as KDM5-C49 and KDM5-C70, respectively) [25], in our cellular high-content assays quantifying changes in the global methylation levels 24 h after addition of the compound. The pyridine carboxylic acid KDOAM-20 is a highly potent KDM5 inhibitor in vitro, whereas the more cell-active ethyl ester derivative KDOAM-21 most likely acts as prodrug, which is hydrolysed in cells to provide KDOAM-20, an approach utilized also in other carboxylic acid-bearing KDM inhibitor scaffolds and 2-OG oxygenase inhibitors (Table 1) [21, 23, 27]. In parallel, we also tested a related control compound, KDOAM-32, in which the core scaffold has been modified to abrogate KDM-binding (Table 1; Additional file 3: Figure S2).

**Table 1:** In vitro and cellular IC50 of selected KDM inhibitors tested

	KDM5A	KDM5B	KDM5C	KDM5D	KDM2A	KDM3A	KDM4A	KDM4B	KDM4C	KDM6B
	IC_50_ (µM)	IC_50_ (µM)	IC_50_ (µM)	IC_50_ (µM)	IC_50_ (µM)	IC_50_ (µM)	IC_50_ (µM)	IC_50_ (µM)	IC_50_ (µM)	IC_50_ (µM)
KDOAM-20	0.010 ± 0.0030 (6)	0.007 ± 0.0030 (12)	0.019 ± 0.0050 (10)	0.019 ± 0.0040 (6)	2.2 ± 0.37 (2)	1.2 ± 0.91 (4)	0.67 ± 0.23 (2)	0.099 (1)	0.19 ± 0.081 (6)	1.4 ± 0.45 (2)
In vitro										
KDOAM-20	ND	20	ND	ND	ND	ND	ND	ND	ND	ND
In cells										
KDOAM-21	0.068 ± 0.010 (2)	0.094 ± 0.065 (6)	0.17 ± 0.073 (6)	ND	19 ± 10 (2)	2.6 ± 1.3 (4)	11 ± 4.5 (2)	2.5 (1)	2.8 ± 1.3 (4)	11 ± 4.6 (2)
In vitro										
KDOAM-21	4.8	0.7	3.1	4	NA	NA	70	10*	17.9	NA
In cells										
KDOAM-32	ND	>10 (2)	ND	ND	ND	ND	ND	ND	ND	ND
In vitro										
KDOAM-32	>100	>100	>100	>100	>100	>100	>100	>100	>100	>100
In cells										
KDIPP15	0.040 ± 0.016 (2)	0.063 ± 0.028 (2)	0.26 ± 0.039 (2)	0.23 ± 0.039 (2)	>100 (2)	>100 (2)	3.9 ± 1.4 (3)	1.4 ± 0.47 (3)	5.1 ± 0.062 (4)	>100 (2)
In vitro										
KDIPP15	ND	20.7	ND	ND	ND	ND	ND	ND	ND	ND
In cells										
KDIPP51 (CCT364883)	0.015 ± 0.0041 (4)	0.014 ± 0.0046 (4)	0.024 ± 0.0044 (4)	0.024 ± 0.0038 (4)	8.6 ± 5.9 (2)	6.0 ± 3.9 (4)	0.126 ± 0.013 (2)	0.050 ± 0.0038 (2)	0.10 ± 0.031 (4)	44 ± 17 (3)
In vitro										
KDIPP51 (CCT364883)	ND	39.9^#^	ND	ND	ND	ND	ND	ND	ND	ND
In cells										
CPI-455	0.16 ± 0.068 (2)	0.26 ± 0.064 (2)	7.8 ± 1.6 (2)	ND	23 ± 3.5 (2)	>100 (2)	31 (1)	44 (1)	11 ± 2.3 (2)	>100 (2)
In vitro										
CPI-455	ND	60	ND	ND	ND	ND	ND	ND	ND	ND
iN cells										
CCT365599	ND	0.023(2)	0.065(2)	ND	12.9^c^	5.3^c^	0.102 ± 0.058	0.031 ± 0.012	ND	15% at 100 µM^c^
In vitro^a^										
CCT365599	ND	>50	ND	ND	ND	ND	10	ND	ND	ND
In cells										
CCT366293	ND	>10^c^	>10^c^	ND	ND	ND	>10 (2)	>10 (2)	ND	ND
In vitro^a^										
CCT366293	ND	>100	ND	ND	ND	ND	>100	ND	ND	ND
In cells										
IOX1	ND	20.6 ± 9.0 (10)	30.4 ± 13.2 (13)	ND	5 ± 3.8 (4)	0.2 ± 0.22 (30)	0.5 ± 0.4 (2)	ND	3.5 ± 2.9 (16)	1.2 ± 1.3 (55)
In vitro^a^										
IOX1	ND	62	ND	ND	ND	ND	ND	ND	ND	ND
In cells										
JIB-04 E	ND	0.561 (1)	0.622 ± 0.05 (2)	ND	1.2 ± 0.01 (3)^d^	0.65 ± 0.05 (3)	ND	ND	0.62 ± 0.02 (2)	1.04 ± 0.6 (4)
In vitro^a^										
JIB-04 E	ND	3	ND	ND	ND	ND	ND	ND	ND	ND
In cells										
JIB-04 Z	ND	7.01 (1)	>10 (3)	ND	ND	>10 (3)	ND	ND	>10 (2)	>10 (4)
In vitro										
JIB-04 Z	ND	>100	ND	ND	ND	ND	ND	ND	ND	ND
In cells										

In vitro

IC50 values ± SD (number of replicates) determined by the AlphaScreen assay

^a^In vitro biochemical profiling was previously reported [11]

^b^Biochemical assay protocols as previously reported [11]. CCT366293 displays assay interference above 10 μM

^c^Results are from a single determination

^d^Results are determined by RapidFire under low iron condition (0.2 mM)

In cells

EC50 values were determined by IF assay

Results are mean values of at least 3 independent experiments unless indicated otherwise

* Results from 2 independent experiments

^*#*^Highest concentration was excluded from calculation

HeLa cells transiently overexpressing WT KDM5B were treated with the ester KDOAM-21 for 24 h. At the highest compound concentration tested, the KDM5 enzymes were completely inhibited and H3K4me3 levels reached that of the cells transfected with the MUT KDM5 enzyme (Fig. 2a (i–iii), b). In line with the in vitro biochemical potency of the parent carboxylic acid (KDOAM-20), KDOAM-21 showed potent inhibition of H3K4me3 demethylation caused by overexpressed KDM5B with an EC_50_ < 1 µM. Surprisingly, KDOAM-20 also showed activity against KDM5B in cells with an EC_50_ of 20 µM despite having low cell permeability in Caco-2 assays (Additional file 4: Table S2). The other KDM5 family members tested (KDM5A, KDM5C and KDM5D) were also potently inhibited by KDOAM-21 with EC_50_ values in the 3–5 μM range in line with the measured in vitro potency of the parent carboxylic acid KDOAM-20 (Fig. 2b; Table 1), but showed little inhibition upon treatment with KDOAM-20 consistent with poor cell penetrance of the parent carboxylic acid (Additional file 4: Table S2). KDOAM-20/21 also showed inhibition of the KDM4 family, which was assessed by increase of the H3K9me3 mark. KDM4B was inhibited most potently with an EC_50_ value of 10 μM for KDOAM-21 (Fig. 2c; Table 1). For the KDM4 subfamily members tested, we also observed inhibition of the overexpressed enzymes by the compounds within the same potency rank order as observed in enzyme kinetic assays (Fig. 2c; Table 1; Additional file 4: Table S2). None of the inhibitors showed activity on members of the other demethylase families tested (KDM3A activity on H3K9me2, KDM6B activity on H3K27me3) (Fig. 2c; Table 1) consistent with the weak in vitro enzymatic potency of these compounds versus the respective demethylase assays.

Interestingly, at higher KDOAM-20/21 inhibitor concentrations, a dose-dependent effect on the H3K4me3 mark was observed in cells overexpressing MUT KDM5. This effect was not seen in cells expressing WT or MUT KDM5 treated with the control compound KDOAM-32, possibly indicating that the observed effects were either due to inhibition of the endogenous enzyme(s) or due to a nonspecific effect of KDOAM-21 and KDOAM-20 (Fig. 2b). We observed no toxicity as measured by cell count using HeLa cells at any of the measured concentrations, indicated by a constant number of cells assessed in the high-content screen (Additional file 5: Figure S3). In order to further study inhibition of endogenous H3K4me3 levels, nontransfected HeLa cells were treated with KDOAM-20, KDOAM-21 and the inactive control KDOAM-32 at the same concentrations used in assays where the protein was ectopically expressed. H3K4me3 was monitored after 72 h by IF staining. Observed EC_50_ (sub-μM potency for KDOAM-21, ~50 μM for KDOAM-20 and no inhibition observed for KDOAM-32) were in good agreement with EC_50_ values measured using the overexpressed enzyme (Fig. 2d; Table 1). Similar results were obtained after treatment of nontransfected HeLa cells for 48 h, whereas at 24 h the observed EC_50_ values were slightly lower (Hatch et al. unpublished results). We therefore have established a robust panel of cell assays to test inhibitors for the Jumonji family of KDMs.

**Fig. 3:**
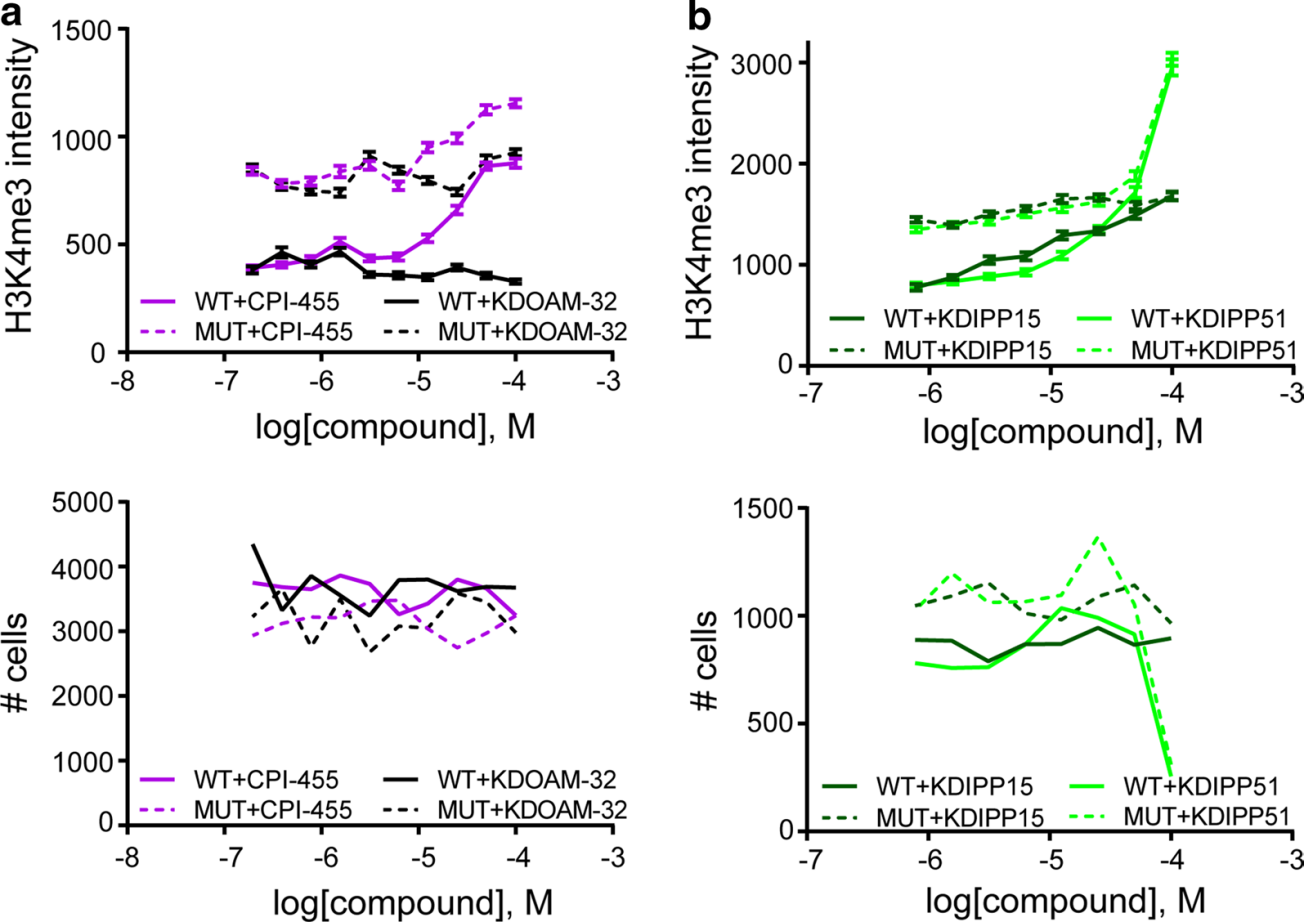
Effect of different KDM5 inhibitors on histone tail methylation status in HeLa cells. **a** Immunofluorescence assays of cells transfected with KDM5B and treated with the indicated inhibitors CPI-455 and KDOAM-32, respectively. Effect on H3K4me3 methylation is shown to be dose dependent in the *upper panel*. Number of cells in 20 fields, based on DAPI staining, is shown in the *lower panel*, **b** immunofluorescence assays of cells transfected with KDM5B and treated with inhibitors KDIPP15 or KDIPP51, as indicated. The dose-dependent effect on H3K4me3 methylation is shown in the *upper panel*. Number of cells in 20 fields, based on DAPI staining, is shown in the *lower panel*

Treatment of cells with inhibitors of the recently disclosed cell-penetrant cyanopyrazole class such as CPI-455 [28, 29] (Table 1; Additional file 3: Figure S2; Additional file 4: Table S2) resulted in a dose-dependent increase of the H3K4me3 mark (Fig. 3a). An increase in methylation in both WT and MUT KDM5-expressing cells was observed, which was even more pronounced than noted with KDOAM-21, as described above. No reduction in cell numbers was observed after treatment with this inhibitor, indicating that the compound is not toxic at the exposures examined (Fig. 3a). We hypothesized that the observed CPI-455-dependent increase of the H3K4me3 mark in cells overexpressing the mutant KDM5B protein was due to compound-mediated inhibition of the endogenous KDM5 enzyme(s) as observed for the KDOAM series.

**Fig. 4:**
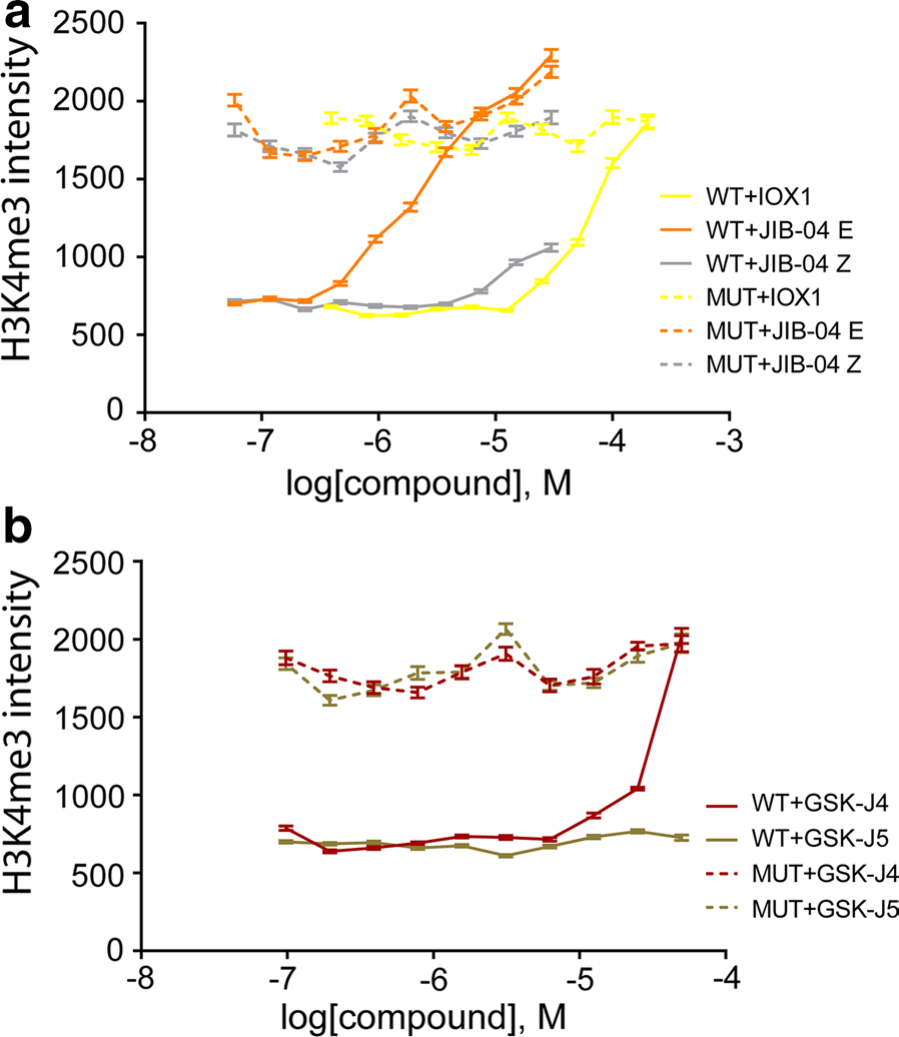
Effect of various KDM inhibitors described in the literature on KDM5B-mediated H3K4me3 methylation in HeLa cells. **a** Effect of Pan-KDM inhibitors IOX1 [30] and JIB-04 E as well as inactive control compound JIB-04 Z [16] on KDM5B-mediated H3K4me3 demethylation, **b** effect of KDM6A/B inhibitor GSK-J4 and inactive control compound GSK-J5 [21, 31] on KDM5B-mediated H3K4me3 demethylation. Data represent the average and SEM of at least 100 cells

In the weakly acidic pyridopyrimidinone series [13] (Table 1; Additional file 3: Figure S2), we observed dose-response cellular activity in the KDM5B IF assay. Treatment with KDIPP51 (CCT364883) resulted in a marked spike indicating increased methylation levels in both WT and MUT KDM5B-expressing cells at the highest inhibitor concentration (Fig. 3b). This spike in methylation levels correlates with a steep drop in cell numbers (Fig. 3b). The potent inhibitor KDOAM-21 demonstrated that KDM5 inhibition per se is not toxic to HeLa cells, so the observed cell death is unlikely to be solely due to inhibition of KDM5 demethylation activity. Cell permeability for both compounds is good and better for KDIPP15 compared to KDIPP51 (Additional file 4: Table S2). We therefore propose that the observed spike in H3K4 methylation is most likely due to nonspecific off-target effects of KDIPP51 and/or is due to the simultaneous inhibition of several KDMs. In cells overexpressing KDM5B and treated with the promiscuous KDM inhibitors IOX1 [30] and JIB-04 [16], we only observed cell toxicity at high concentrations (Fig. 4a; Additional file 6: Figure S4). Both of these inhibitors elicited a dose-dependent increase of H3K4me3 in cells transfected with WT KDM5B. In addition, treatment with the broad-spectrum KDM inhibitor JIB-04 at concentrations above ~20 µM resulted in an increase of H3K4me3 in cells expressing either the WT or the MUT enzyme similar to what was observed with KDIPP51 in cells overexpressing KDM5B (Table 1; Fig. 4a) and accompanied by a reduction in cell number (Additional file 6: Figure S4).

The KDM6A/B inhibitor GSK-J4 [21] is reported to also inhibit KDM5 at higher concentrations in vitro [31]. We tested GSK-J4 together with its inactive control compound GSK-J5 and also observed an increase of the H3K4me3 mark upon treatment with the active KDM6A/B inhibitor, but not the inactive control. These two compounds showed only minor toxicity at the highest concentrations used, which is well above the effective doses used for KDM6A/B inhibition (~1 µM) (Fig. 4b; Additional file 6: Figure S4). Assessment of inhibitors in the high-content assay therefore needs careful consideration of all parameters, WT and MUT data. In addition, steep-slope increases of histone methylation mark are often accompanied by a reduction in cell number pointing to cell toxicity of the compound. The results imply that simultaneous inhibition of several KDMs does not seem to be tolerated at higher compound concentrations by the tested cells.

**Fig. 5:**
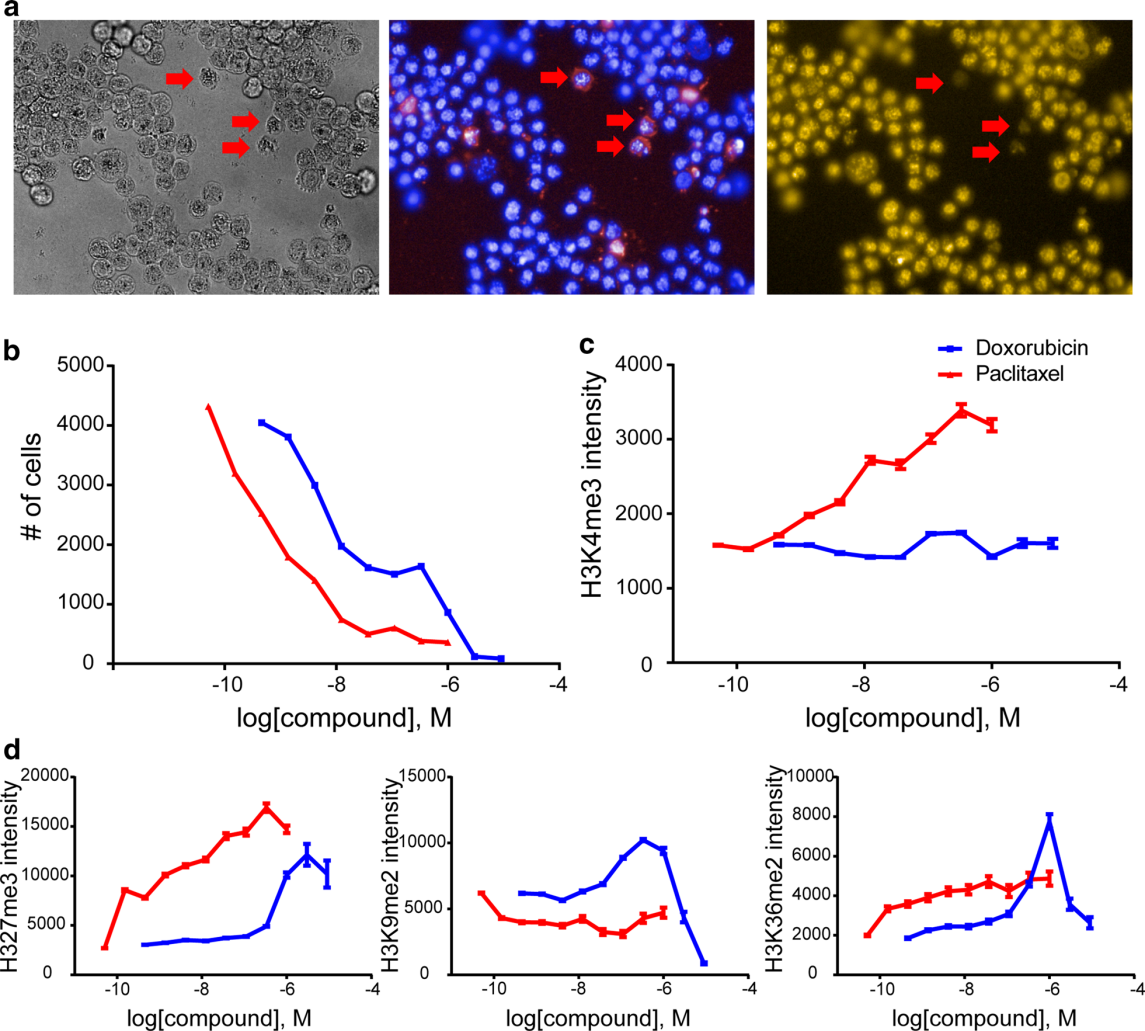
Apoptosis induced by different mechanisms is accompanied by methylation mark changes. **a** Images of HeLa cells treated with 100 nM paclitaxel for 24 h and stained with DAPI and a specific antibody for H3K4me3. The phase contrast image is shown to the *left*, *blue* DAPI nuclear stain in the *middle* and *yellow* H3K4me3 to the *right*. *Arrows* indicate apoptotic cells lacking the H3K4me3 mark, **b** number of HeLa cells treated with doxorubicin or paclitaxel in a dose-dependent manner based on counting of 12 fields, **c** immunofluorescence assay showing the effect of doxorubicin or paclitaxel treatment on H3K4me3 mark, **d** immunofluorescence assay showing the effect of doxorubicin or paclitaxel treatment on H3K27me3 mark, H3K9me2 mark and H3K36me2 mark, respectively

The observed spike in histone methylation at toxic compound concentrations prompted us to test the effect of agents that cause cytotoxicity by different mechanisms but that are known not to be associated with KDM inhibitor activity (Additional file 7: Table S3). Accordingly, we treated HeLa cells with the DNA intercalator doxorubicin and the microtubule stabilizer paclitaxel and monitored the effect of these cytotoxic agents on H3K4me3 levels (Fig. 5a). Both these compounds induced cell death with the expected EC_50_ values as estimated from the number of cells remaining per field after fixation (Fig. 5b). Surprisingly, paclitaxel also led to a dose-dependent increase of H3K4me3, whereas doxorubicin had no effect on this mark indicating that cell death can be associated with an increase in H3K4me3 without direct inhibition of demethylases (Fig. 5c). We then tested the influence of these two compounds on the other histone marks used to assess KDM activity (Fig. 5d). Both inhibitors had a profound effect on the repressive H3K27me3 mark. Conversely, H3K9me2 levels were not affected by cell death induced by paclitaxel, but showed some increase upon treatment with doxorubicin, although the effects were more variable. Globally, H3K36me2 levels were only marginally affected by cell death induced by either of the two inhibitors but individual cells could show a complete lack of this mark upon treatment (Additional file 8: Figure S5). Similar effects on histone methylation have been noted using other compounds causing cell death like the broad-spectrum kinase inhibitor staurosporine (unpublished observation). None of the three compounds had any specific effect on inhibition of KDM4C, KDM5B or KDM6B in in vitro demethylase assays (Additional file 7: Table S3), indicating that mechanisms inducing cytotoxicity can commonly affect global histone methylation marks.

**Fig. 6:**
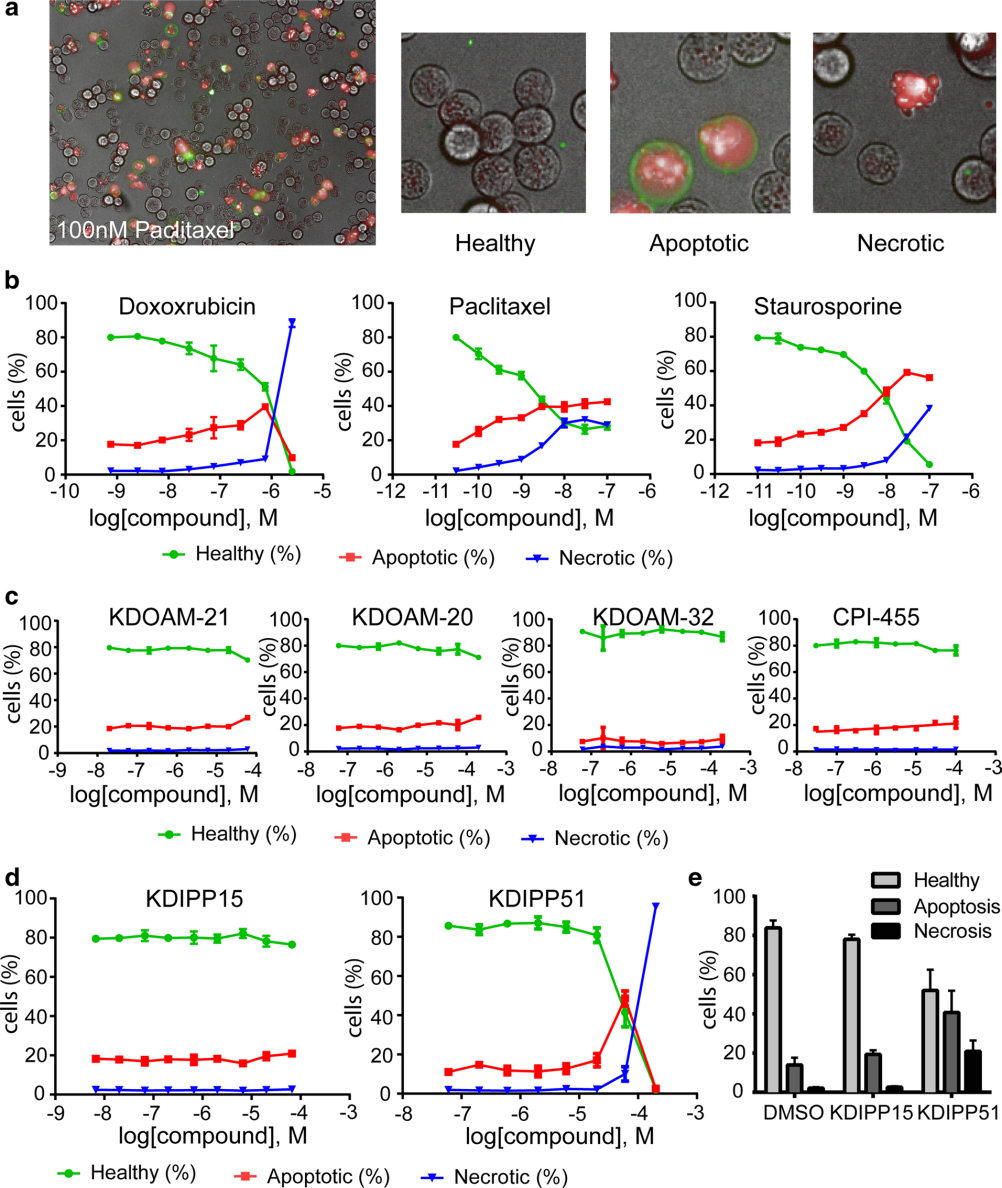
Cytotoxic activity of different KDM inhibitors in live cells. **a** High-content images of HeLa cells treated with 100 nM paclitaxel for 24 h and stained with Annexin V (*green*) and Yo-Pro (*red*). Apoptotic cells were defined as Annexin V positive with or without Yo-Pro 3 uptake; necrotic cells were defined as Yo-Pro 3 positive; and healthy cells were defined as Annexin V and Yo-Pro 3 negative, **b** percentage of healthy, apoptotic and necrotic HeLa cells treated with doxorubicin, paclitaxel or staurosporine in a dose-dependent manner, **c** percentage of healthy, apoptotic and necrotic HeLa cells treated with KDM inhibitors of the KDOAM series  or CPI-455 for 24 h in a dose-dependent manner, **c** immunofluorescence assay showing the effect of doxorubicin or paclitaxel treatment on H3K4me3 mark, **d** percentage of healthy, apoptotic and necrotic HeLa cells treated with compounds of the KDIPP series for 24 h in a dose-dependent manner, **e** percentage of healthy, apoptotic and necrotic HeLa cells treated with KDIPP15 (66 µM) or KDIPP51 (60 µM) for 24 h. Results are shown as mean ± SD from triplicates of two independent experiments

To assess the mode of cell death caused by these compounds and by the tested KDM inhibitors in more detail, we performed a high-content-based triple staining protocol (Fig. 6). Cells were categorized into healthy cells (Hoechst staining only), apoptotic cells defined as Annexin V positive with or without Yo-Pro 3 uptake, or necrotic cells defined by Yo-Pro 3-positive Annexin V negative staining (Fig. 6a) [32]. After 24 h of treatment with doxorubicin, paclitaxel or the pan-kinase inhibitor staurosporine, cell death occurred and was accompanied by the appearance of a predominant apoptotic staining, in line with their known mechanism of action. At higher concentrations the number of necrotic cells increased as monitored by a Yo-Pro 3-positive staining. In contrast, cells treated with DMSO were defined as ‘healthy’ and showed predominantly a negative staining for both Annexin V and Yo-Pro 3 (Fig. 6b). We then tested the different KDM inhibitors to assess their effect on cell viability in more detail. As expected, inhibitors of the KDOAM series (KDOAM-20, KDOAM-21 and KDOAM-32) did not induce cell death at any of the concentrations measured, above the level of DMSO nor did CPI-455 or KDIPP15 (Fig. 6c, d). However, treatment of HeLa cells with KDIPP51 (at 60 µM) for 24 h resulted in 40% apoptotic and 20% necrotic cells as compared to 20% apoptotic and 2% apoptotic cells upon treatment with KDIPP15 (66 µM), similar to what was observed for DMSO-treated cells, indicating that the observed changes in methylation coincide with cell death accompanied by increased apoptosis and necrosis (Fig. 6e). In the same assay, the pan-KDM inhibitors IOX1 and JIB-04 and its inactive control displayed little to no cell toxicity, with only a minor effect of JIB-04 on cellular viability at 30 µM. However, both the KDM6B inhibitor GSK-J4 and its inactive control GSK-J5 (Additional file 9: Figure S6) showed about 60% necrotic cells at 50 µM concentration, which is well above the concentration required to inhibit KDM5/6 activity in cellular assays.

**Fig. 7:**
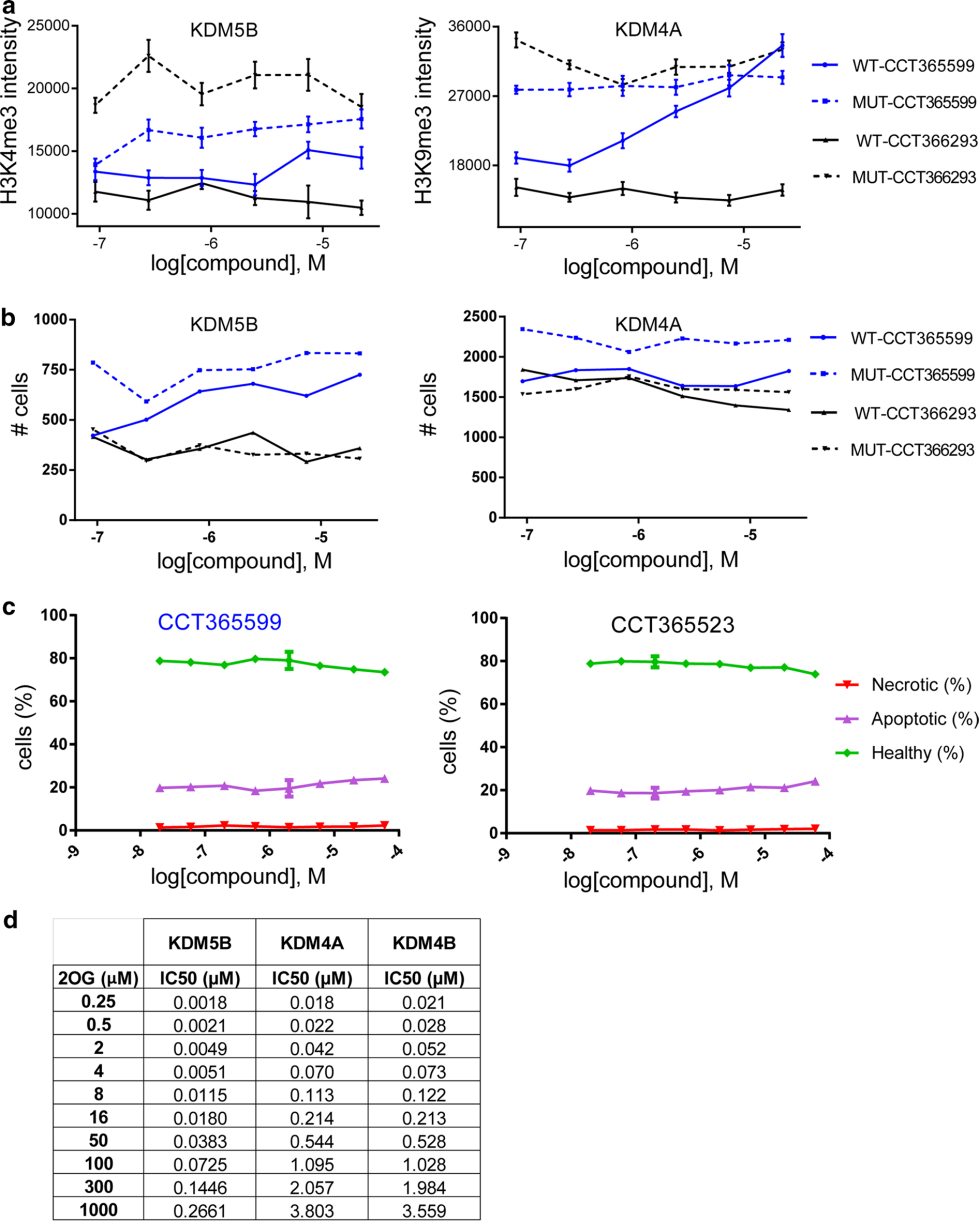
Cytotoxic activity of different KDM inhibitors in live cells and 2-OG dependency of KDM inhibition in vitro. Immunofluorescence assays of cells transfected with KDM5B or KDM4A and treated with the newly developed inhibitors CCT365599 and CCT366293, respectively. Effect on H3K4me3 methylation is shown in the *left panel* in a dose-dependent manner and on H3K9me3 in the *right panel*. Number of cells in 20 fields, based on DAPI staining, is shown in **b**, **c**. Percentage of healthy, apoptotic and necrotic HeLa cells treated with CCT365599 and CCT366293, respectively, in a dose-dependent manner, **d** IC50 values in dependency of different 2-OG concentrations determined in AlphaScreen assay for different KDMs

We have recently described the discovery of cell-penetrant analogues of the pyridopyrimidinone KDIPP51 as dual KDM4/5-subfamily inhibitors [11]. We have used the assays described herein to measure the cellular activity of these compounds and their matched negative control analogues that we report here for the first time. Treatment of HeLa cells with CCT365599 resulted in a concentration-dependent increase of H3K9me3 and H3K4me3 levels, while treatment with the respective negative controls CCT366293 had no effect (Fig. 7a). No cytotoxicity was observed for these compounds at the concentrations used to assess H3K4me3 and H3K9me3 demethylation (Fig. 7b, c), suggesting that in these cases the increase in methylation is due to inhibition of the corresponding demethylase. However, we notice for these compounds and the other compounds tested that there is a drop-off in potency between the biochemical assays and cellular activity ranging from 20- to 300-fold. This effect has been partly attributed to intracellular competition by high concentrations of the endogenous cofactor 2-OG [33]. To examine this, we measured the potency of CCT365599 and CCT365523 in the biochemical assays in the presence of increasing concentrations of 2-OG (Fig. 7d). Consistent with these inhibitors being competitive with the co-substrate, the potency of the compounds shifts in the presence of 2-OG, leading to values in the 3–5 μM range in the presence of physiologically relevant 2-OG concentrations (1000 μM).

## Discussion

Almost all currently available JmjC-KDM inhibitors act via metal chelation and compete with the 2-OG co-substrate for binding at the active site. Poor cellular activity has been a problem; however, cell permeability is not the only reason for the poor cellular activities of the 2-OG competitive KDM inhibitors [11]. The results presented here further inform on the cellular activities of KDM inhibitors. They indicate that the currently used in vitro assays may overestimate the cellular effects due to the use of lower than physiologically relevant 2-OG concentration.

A number of methods have been described to assess cellular activity of KDM inhibitors, each with advantages as well as disadvantages [20]. We have described a panel of cellular assays to assess the direct inhibition of KDMs as well as selectivity of KDM inhibitors in cultured human cells using a high-content-based overexpression system. We assessed several known KDM inhibitors as well as newly described inhibitors and demonstrate the usefulness of structure-matched inactive control compounds to assess effects related to specific KDM inhibition. In addition, the results obtained with overexpression of the MUT protein in combination with assessment of cell numbers provide a useful guide to enzyme inhibition in cells, helping to identify on-target effects. It is now our observation across multiple compounds that, in our hands, a spike in histone mark methylation is characteristic of cytotoxicity. The results obtained using this system are in good agreement with data obtained assaying endogenous demethylases, but offer the additional advantage of distinguishing between a direct effect on KDM inhibition and changes to histone methylation upon induction of cell death.

Cell death observed upon treatment with toxic compounds (e.g. doxorubicin or paclitaxel) resulted in a dose-dependent increase in histone methylation, in particular of the repressive H3K27me3 mark, but also of other marks such as H3K4me3 and H3K9me2. A recent study reported the induction of global changes in histone lysine and arginine methylation and altered expression of lysine demethylases upon treatment of human cancer cells with HDAC inhibitors pointing to a complex network of histone crosstalk [34]. Increased methylation of histone H3K27 associated with apoptosis induced by staurosporine has also been described in osteosarcoma cells, but the study did not examine any changes in dimethyl or trimethyl histone H3K4 and H3K9 [35].

Although not addressed in the current study, one can speculate regarding the underlying physiological mechanisms of these histone mark changes in apoptosis and general cell death. Little is known about changes in histone methylation upon induction of cell death in humans although a global decrease in histone acetylation has been observed during apoptosis [36, 37]. Loss of histone H3 methylation at lysine 4 has, however, been shown to trigger apoptosis in *Saccharomyces cerevisiae* [38]. In melanoma cells, short-term treatment (4 h) with doxorubicin decreased H3K4me3 methylation but did not have an effect on H3K27me3 [39]. Detailed chromatin immunoprecipitation-based assays and gene expression analyses, as well as time-resolved experiments, will be necessary to investigate in more detail the interplay between cell death and chromatin changes addressing questions of specific mechanisms—for example, if cells first activate genes through increased density of H3K4me3 activating marks before increasing repressive marks like H3K27me3 when cell death cannot be avoided.

## Conclusions

In summary, we have developed a series of overexpression IF assays to assess the target engagement of KDM inhibitors in cells. The presented assays cover most major branches of the 2-OG JmjC-KDMs. Nonspecific toxicity of tested inhibitors, in particular those resulting in apoptosis, leads to changes in histone tail modifications unrelated to the expected cellular activity of these compounds. The presented assay provides a suitable method to distinguish between direct and indirect effects of histone methylation.

## Methods

### Cloning

Full-length cDNA for KDM5A (JARID1A, P29375), KDM5B (JARID1B, Q9UGL1, cDNA), KDM5C (JARID1C, P41229, IMAGE 5492114), KDM4A (O75164) containing A482E substitution (natural variant rs586339), KDM4B (JMJD2B, O94953, gift from ICR), KDM4C (JMJD2C, Q9H3R0, 8143862), KDM3A (JMJD1A, Q9Y4C1, 4823253), KDM2A (FBXL11, Q9Y2K7, 5534384) and KDM6B (JMJD3, O15054, cDNA) were amplified by PCR from either a MGC clone or commercial cDNA source or received as a gift from collaborators and cloned into pDONR-221 vector using Gateway BP reaction producing Gateway entry clones.

To produce catalytically inactive KDMs residues involved in iron coordination were substituted for alanine (KDM5A H483A/E485A, KDM5B H499A/E501A, KDM5C H513A/E515A, KDM5D H510A/E512A, KDM4A H188A/E190A, KDM4B H189A/E191A, KDM4C H190A/E192A, KDM3A H1120Y, KDM2A H212A/D214A and KDM6B H1390A/E1392A). Mutations were introduced into full-length KDM Gateway entry clones using 15 cycles QuikChange II PCR protocol (Agilent Technologies).

Mammalian expression constructs encoding for N-terminal 3*FLAG potencies were constructed using the Gateway LR recombination reaction between pCDNA5-FRT/TO-3FLAG destination vector [40] and the wild-type or mutated KDM Gateway entry clone.

All constructs described are available upon request.

### Cell culture/transfection for 24-h IF assay

HeLa cells (ATCC) were cultured at 37 °C and 5% CO_2_ in Minimal Essential Media—Eagle (Sigma-Aldrich, UK) supplemented with 10% heat-inactivated foetal calf serum (FCS) (Gibco, UK), and 1% l-glutaMAX (Gibco, UK) in T75 flasks (Sarstedt, UK) and passaged regularly with TrypLE (Life Technologies, UK) before cells reached confluence. On day 0, 7000 cells per well were plated on a 96-well clear bottom CellCarrier plate (PerkinElmer, UK). The next day the cells were transfected with Lipofectamine 3000 (ThermoFisher Scientific, UK) following the manufacturer’s instructions. Briefly, 0.1 µg of plasmid DNA (encoding for the respective demethylase) and 0.15 µL of Lipofectamine 3000 were diluted in separate tubes containing 5 µL OptiMEM. 0.15 µL of P3000 was added to the tube with the DNA. The two solutions were then mixed gently and allowed to incubate for 5 min at room temperature. Next, the DNA-lipid complex was added to the cells for 4 h at 37 °C and 5% CO_2_.

Compound dilutions were made in a 96-well PCR plate (Starlab, UK). The culture medium was supplemented with 0.4% DMSO so that the solvent concentration was maintained throughout. Compounds were serially diluted at a 1:2 or 1:3 dilution ratio. Next, the cell culture plate was removed from the incubator and the medium was gently aspirated and replaced with the fresh medium containing diluted compounds.

After 24 h of compound incubation, cells were stained using the following protocol. First, the cells were washed once with Phosphate Buffered Saline (PBS) (Sigma-Aldrich, UK) and fixed for 20 min at room temperature using formaldehyde diluted in PBS to 4% w/v. Next, cells were rinsed once with PBS and permeabilized for 5 min at room temperature using TritonX-100 diluted in PBS at 0.5% v/v. Then cells were rinsed once with PBS and blocked for 30 min at room temperature using 3% v/v foetal calf serum diluted in PBS. This mixture was then replaced with the appropriate primary antibodies for the histone mark and FLAG-tag diluted in blocking solution and left on overnight at 4 °C. The following day, cells were rinsed three times with PBS and stained with the secondary antibody for 1 h at room temperature. Before imaging, cells were rinsed once with PBS, stained with DAPI (LifeTechnologies, UK) for 5 min and rinsed again with PBS at room temperature.

### Endogenous IF assay

The experimental set-up was similar to the 24-h assay except for the following modifications. Cells were cultured in OptiMEM (LifeTechnologies, UK) and supplemented with 0.5% foetal calf serum (FCS) (Gibco, UK) and 1% l-glutaMAX (Gibco, UK). HeLa cells were seeded at a density of 800 cells per well on day 0. The next day, a compound dilution plate (diluted in OptiMEM with supplements) was made and the medium from the day before replaced. The cells were incubated for 72 h at 37 °C and 5% CO_2_. After fixation, the immunofluorescence protocol was similar as the 24-h assay with the omission of the FLAG-tag antibodies (Table 2).

**Table 2:** Antibody specifications for each demethylase IF assay

Demethylase	Histone mark	Primary antibody for histone mark (cat. no.)	Dilution	Secondary antibody for histone mark (cat. no.)	Dilution	Primary antibody for FLAG-tag (cat. no.)	Dilution	Secondary antibody for FLAG-tag (cat. no.)	Dilution
KDM3A	H3K9me2	Abcam (ab1220)	1:500	Alexafluor 488 (MG2A20)	1:500	F7425	1:500	A-11011	1:500
KDM4A/B/C	H3K9me3	Abcam (ab8898-100)	1:500	Alexafluor 488 (A-11070)	1:500	F3165	1:500	A21124	1:500
KDM6B	H3K27me3	Millipore (07-449)	1:500	Alexafluor 488 (A-11070)	1:500	F3165	1:500	A21124	1:500
KDM5A/B/C	H3K4me3	Diagenode (C15410003-50)	1:500	Alexafluor 488 (A-11070)	1:500	F3165	1:500	A21124	1:500

### Widefield fluorescence microscopy

Transfection of cells was assessed on a Zeiss AxioObserver Z1 inverted fluorescence microscope fitted with an Axiocam 506 monochrome camera, Colibri.2 LED system (385, 475, 561, 647 nm), 10× 0.45 N.A. and 20× 0.8 N.A. Plan Apochromat objectives. The system PC ran Windows 7 Ultimate 64-bit and had an Intel Core i5-2500 @3.30 GHz processor with 8 GB RAM and an integrated video card. The microscope was controlled with ZEN Blue.

### High-content analysis

Immunofluorescence images of 20 fields (if not indicated otherwise) were captured through a 20× objective on a PerkinElmer Operetta or the GE IN Cell 6000 using the appropriate filters and light sources for exciting DAPI, Alexafluor 488, and Alexafluor 568 or Alexafluor 647. The images were imported into Columbus (PerkinElmer) for further analysis. From the DAPI channel, a binary mask was created to exclude individual nuclei smaller than 100 µm^2^ and those overlapping the edge of the field. The remaining nuclei were used for further analysis. The average nuclear intensity of the anti-Flag staining (Alexafluor 568 or Alexafluor 647) was measured, and then the intensity of the anti-methylated histone mark (Alexafluor 488) was analysed only in those cells that strongly overexpressed the FLAG-tagged demethylase. The total number of nuclei was used as a measure of cell survival and to calculate transfection efficiency. For the endogenous assay, the intensity of Alexafluor 488 from all cells in 9–10 fields was used for analysis. The average intensity and standard deviation of Alexafluor 488 cells was measured for each well and plotted in a dose-dependent manner in Prism (GraphPad, USA).

### Hoechst 33342/Yo-Pro 3/Annexin triple staining and live cell death pattern analysis

Cells exposed to the inhibitors for 24 h were stained with Hoechst 33342 (1 µM), Yo-Pro 3 (1 µM) and Annexin V (0.3 μL per well) for 1 h. Cellular fluorescence was measured using the IN Cell 6000 (GE) using the following set-up parameters: bright-field transmitted light at 50% for 15 ms; Hoechst 33342 was excited by 35-ms exposure(Ex 360–400 nm/Em 410–480 nm), Yo-Pro 3 by 35-ms exposure(Ex 560–580 nm/Em 650–760 nm) and Annexin V (Alexa 488) by 45-ms exposure(Ex 460–490 nm/Em 500–550 nm). All the generated data were analysed using the IN Cell Analyser software, and three categories were distinguished: healthy cells, apoptosis and necrosis—calculated as percentage of each class for every concentration used.

### Caco-2 permeability

*P*_app_ (apparent permeability) was determined in the Caco-2 human colon carcinoma cell line essentially as described in [11].

### AlphaScreen

IC_50_ values were determined as previously described [11]. The following amendments were made to the protocol for carrying out 2-OG competition assays.

For each compound, a 10 mM stock concentration in 100% DMSO was used. Compounds (100 nL) were dispensed with an ECHO^®^ 550 acoustic dispenser (Labcyte Inc™, Sunnyvale, CA, USA) to generate 10–12 pt dilution curves directly into 384-well Proxiplates (#6008289, PerkinElmer, Waltham, MA, USA) to give final assay concentrations in the range 0.00015–30 µM in 2%(v/v) DMSO where appropriate. Compounds were pre-incubated with their enzymes (2.5 µL, 1.5 nM KDM4A, 0.5 nM KDM4B or for 2 nM KDM5B final assay concentration) for 10 min at room temperature before the addition of their peptide substrates (2.5 µL).

Peptide substrates (60 nM ARTKQTARK(me3)STGGKAPRKQLA-GGK-biotin for KDM4A and KDM4B or 200 nM ARTK(me3)QTARKSTGGKAPRKQLA-GGK-biotin for KDM5B) were made up in peptide buffer consisting of HEPES (50 mM, pH 7.5), BSA (0.1%), sodium ascorbate (200 µM), ammonium iron(II) sulphate hexahydrate (2 µM for KDM4A/KDM4B and 10 µM for KDM5B), Tween 20 (0.01%) and 11 2-OG concentrations ranging from 2000 to 0.5 µM. Peptide and buffer components were diluted twofold upon the addition to the compound plate containing enzyme in assay buffer [HEPES (50 mM, pH 7.5), BSA (0.1%) and Tween 20 (0.01%)]. The plate was sealed, then centrifuged at 1000 rpm for 1 min before being left for 15 min at room temperature. The reaction was stopped with the addition of 2.5 µL EDTA (30 mM EDTA in water for KDM4A and KDM4B or 30 mM EDTA, 1600 mM NaCl, 50 mM HEPES pH 7.5 and 0.01% Tween 20 for KDM5B) before the addition of 2.5 µL AlphaScreen detection reagents and antibody appropriate for the substrate being detected (mouse IgG beads (#6760606M, PerkinElmer) and anti-dimethyl H3K9 antibody (#Ab1220, Abcam) for KDM4A and KDM4B and Protein A beads (#6760617M, PerkinElmer) and anti-dimethyl H3K4 antibody (#9725S, Cell Signalling Technology) for KDM5B). AlphaScreen beads and antibody mixes were prepared and pre-incubated for 1 h prior to addition to the plate. Plates were left for 1.5 h in the dark before being read on the EnVision^®^ Multilabel Reader (PerkinElmer Life Sciences). All assays were run at a final assay reaction volume of 5 µL and a total assay volume of 10 µL.

Data were normalized to the controls without 2-OG and DMSO wells containing all reagents as the totals. IC_50_ values at each 2-OG concentration were determined using a nonlinear regression fit of the log (inhibitor) versus response with variable slope equation in GraphPad Prism 6.0.

### Chemical synthesis

Additional file 10 Methods.

## Additional files

**Additional file 1: Figure S1.** Ribbon structure of KDM4A based on pdb: 2OQ6. Shown are residues involved in iron coordination with mutated amino acids annotated in black.

**Additional file 2: Table S1.** KDMs and mutant KDMs for which assays are reported including histone mark assessed.

**Additional file 3: Figure S2.** Chemical structure of the KDM inhibitors used in the study.

**Additional file 4: Table S2.** Cell permeability data of compounds tested.

**Additional file 5: Figure S3.** Effect of KDOAM compounds on HeLa cell number transfected with different KDMs. The number of cells in 20 fields, based on DAPI staining, is shown.

**Additional file 6: Figure S4.** Effect of various compounds on HeLa cell number transfected with different KDMs. The number of cells in 20 fields, based on DAPI staining, is shown.

**Additional file 7: Table S3.** Effect of Doxorubicin, Paclitaxel and Staurosporine on KDM activity measured in in vitro KDM assay.

**Additional file 8: Figure S5.** Cell images of HeLa cells treated with 5 µM Doxorubicin or 100 nM Paclitaxel and stained with an anti H3K36me2 antibody.

**Additional file 9: Figure S6.** Percentage of healthy, apoptotic and necrotic HeLa cells treated with KDM inhibitors and negative controls for 24 h in a dose-dependent manner.

**Additional file 10.** Methods and chemical synthesis.

## Abbreviations

KDM: histone lysine demethylase
JmjC: Jumonji C
2-OG: 2-oxoglutarate
IF: immunofluorescence
HCA: high-content assay
