# Supplementary figures and images for: Assessing histone demethylase inhibitors in cells: lessons learned
Source: Epigenetics Chromatin. 2017 Mar 1;10:9. doi: 10.1186/s13072-017-0116-6 (PMC5333395; doi:10.1186/s13072-017-0116-6)

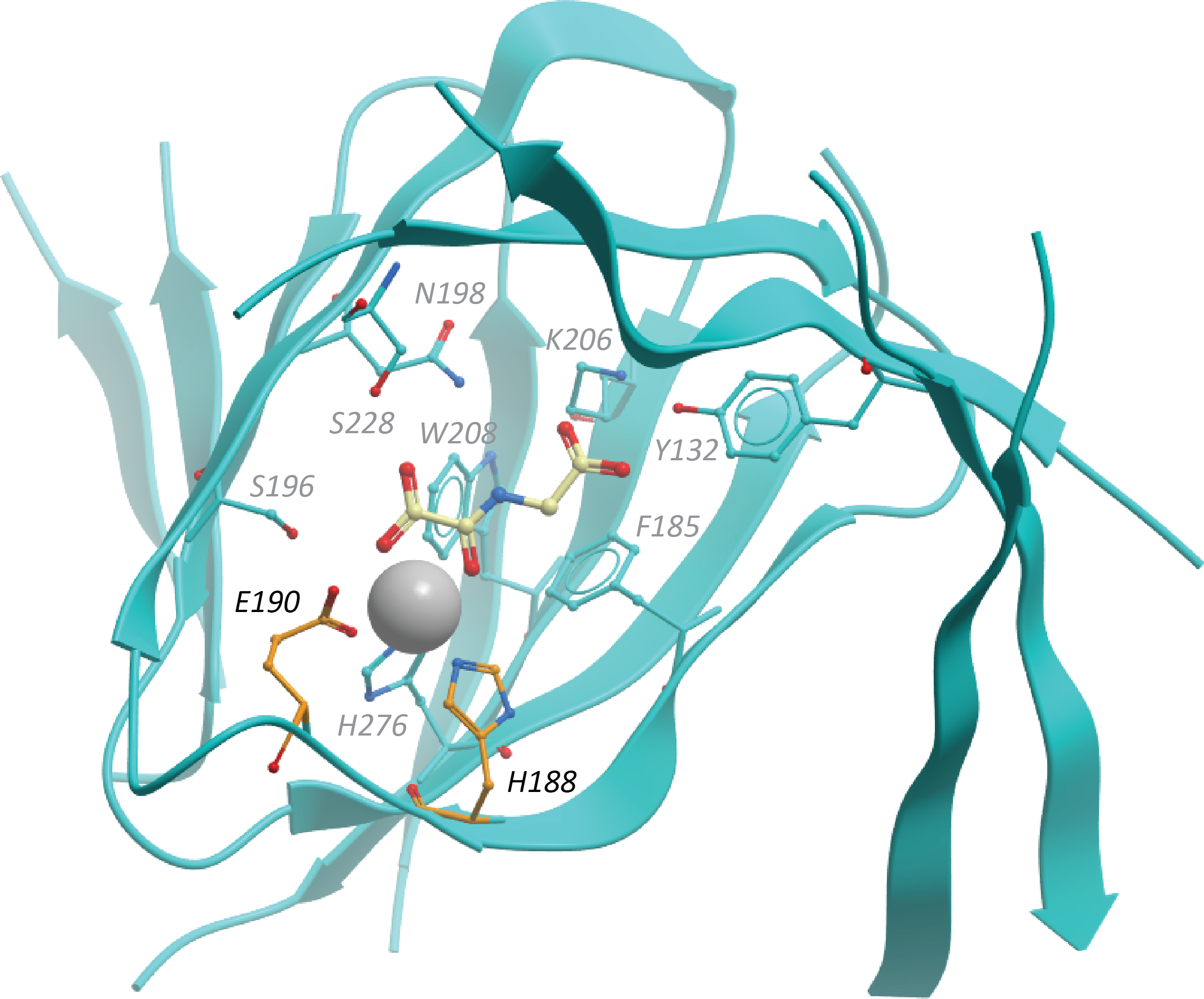

Supplement: Supplementary file 1 — Additional file 1: Figure S1. Ribbon structure of KDM4A based on pdb: 2OQ6. Shown are residues involved in iron coordination with mutated amino acids annotated in black. [file 13072_2017_116_MOESM1_ESM.png]

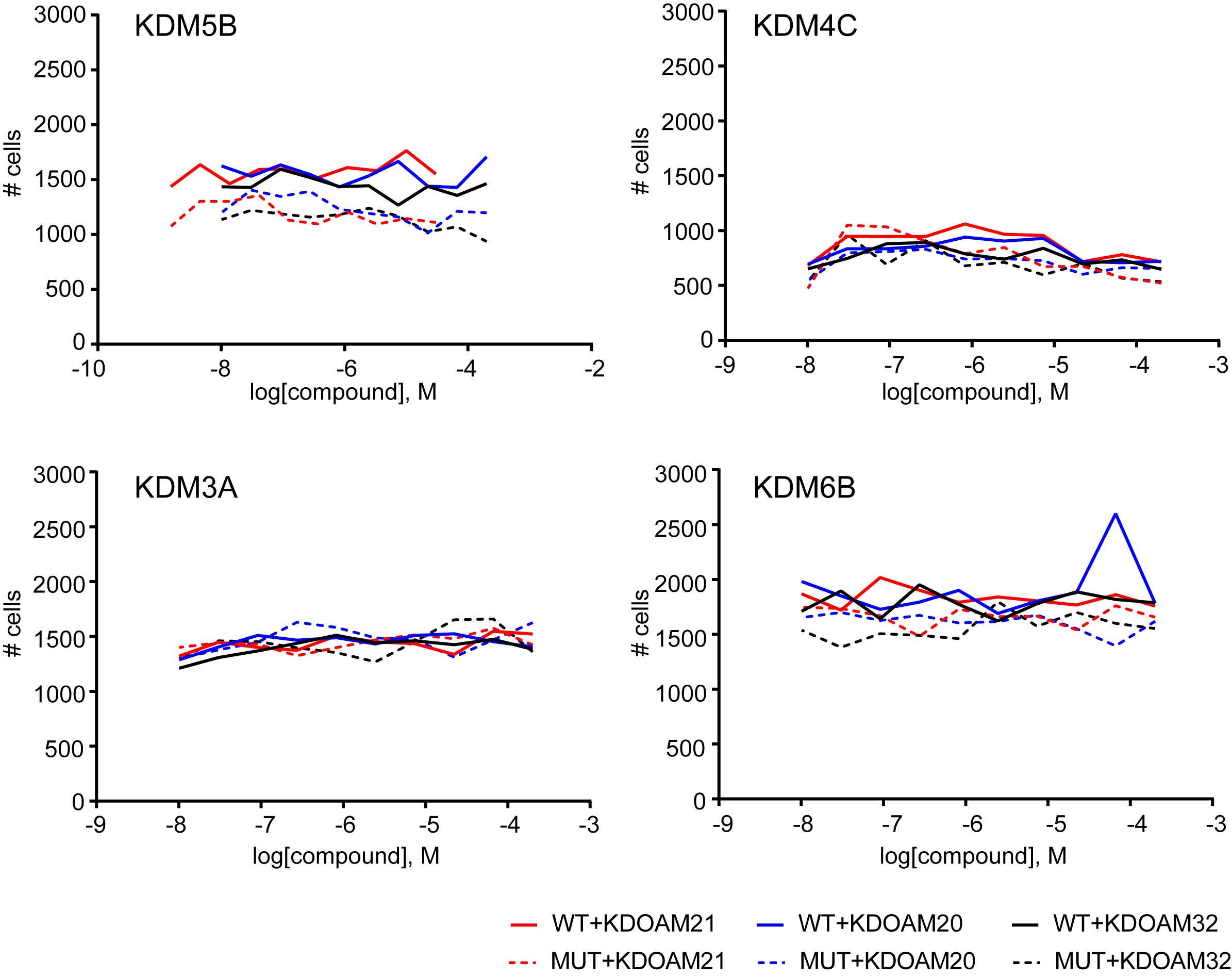

Supplement: Supplementary file 5 — Additional file 5: Figure S3. Effect of KDOAM compounds on HeLa cell number transfected with different KDMs. The number of cells in 20 fields, based on DAPI staining, is shown. [file 13072_2017_116_MOESM5_ESM.png]

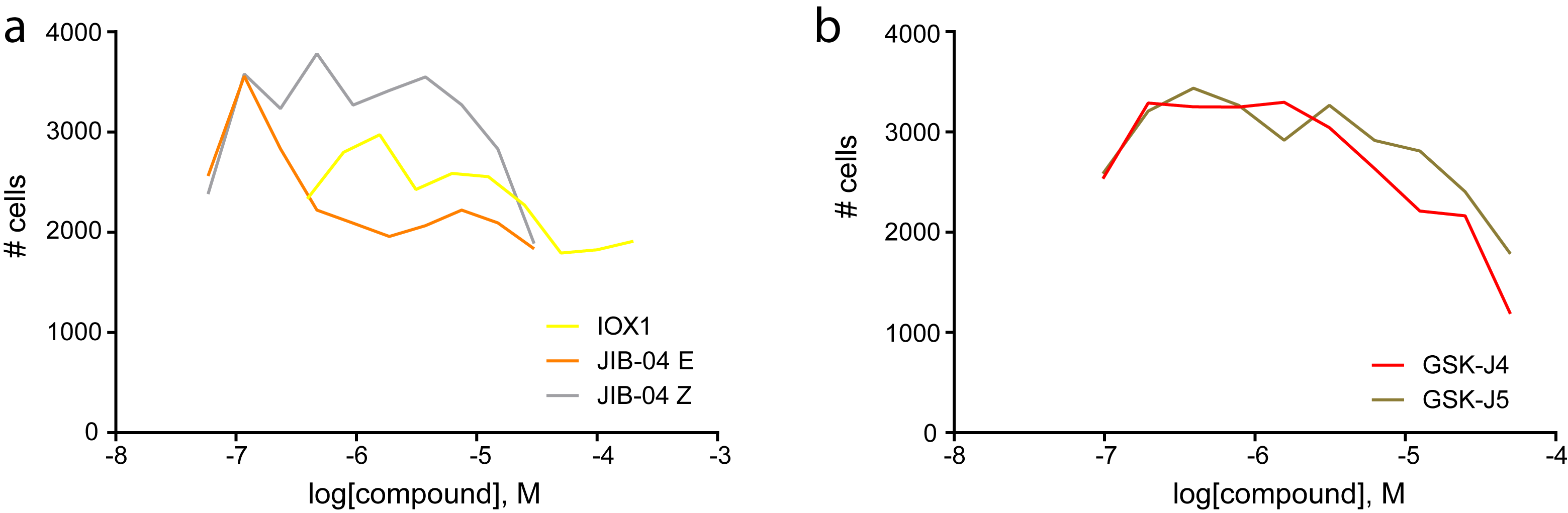

Supplement: Supplementary file 6 — Additional file 6: Figure S4. Effect of various compounds on HeLa cell number transfected with different KDMs. The number of cells in 20 fields, based on DAPI staining, is shown. [file 13072_2017_116_MOESM6_ESM.png]

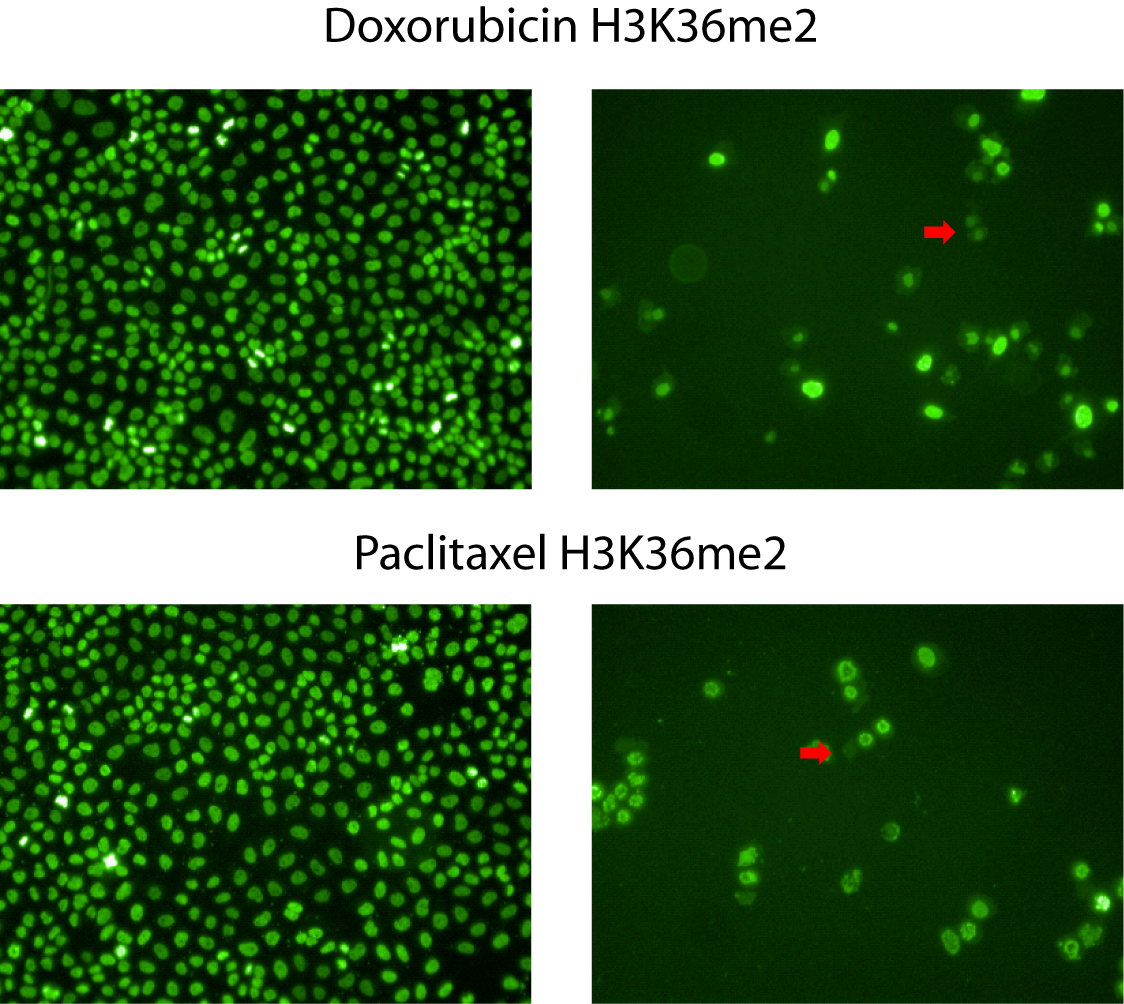

Supplement: Supplementary file 8 — Additional file 8: Figure S5. Cell images of HeLa cells treated with 5 µM Doxorubicin or 100 nM Paclitaxel and stained with an anti H3K36me2 antibody. [file 13072_2017_116_MOESM8_ESM.png]

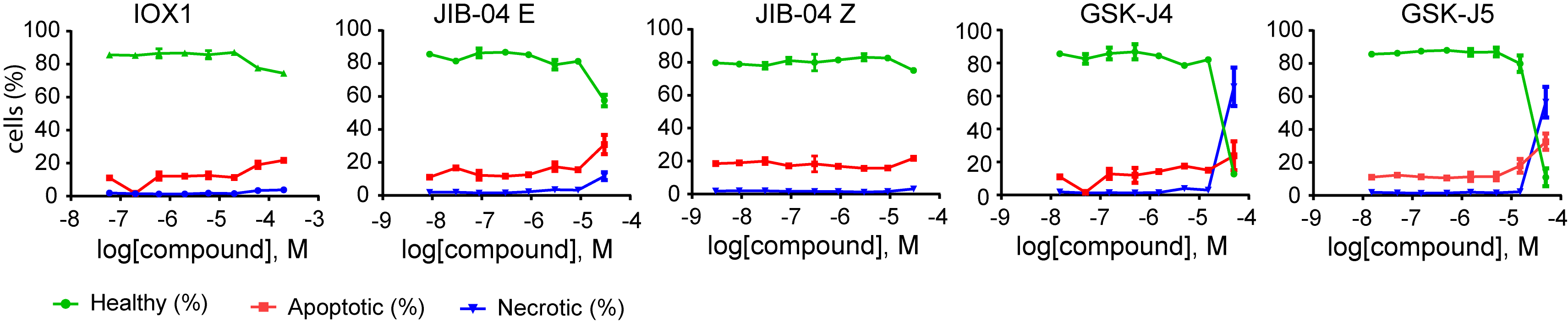

Supplement: Supplementary file 9 — Additional file 9: Figure S6. Percentage of healthy, apoptotic and necrotic HeLa cells treated with KDM inhibitors and negative controls for 24 h in a dose-dependent manner. [file 13072_2017_116_MOESM9_ESM.png]
