# Supplementary material for: Assessing histone demethylase inhibitors in cells: lessons learned
Source: Epigenetics Chromatin. 2017 Mar 1;10:9. doi: 10.1186/s13072-017-0116-6 (PMC5333395; doi:10.1186/s13072-017-0116-6)
Supplement: Supplementary file 10 — Additional file 10. Methods and chemical synthesis. [file 13072_2017_116_MOESM10_ESM.docx]

Supplementary Methods

**Chemistry:** Commercially available starting materials, reagents and anhydrous solvents were used as supplied. Flash column chromatography was performed using Merck silica gel 60 (0.025 – 0.04 mm). Thin layer chromatography was performed using Merck Millipore TLC silica gel 60 F_254_ aluminium sheets and visualised by UV (254 & 280 nm), iodine and KMnO_4_. Column chromatography was also performed on a FlashMaster personal unit using isolute Flash silica columns or a Biotage Isolera purification system using Biotage KP-SNAP cartridges. Ion exchange chromatography was performed using acidic Isolute Flash SCX-II cartridges. ^1^H NMR spectra were recorded using either a Bruker Avance-500 or Bruker Avance-400 NMR machine. Samples were prepared as solutions in a deuterated solvent and referenced to the appropriate internal non-deuterated solvent peak or tetramethylsilane. Chemical shifts were recorded in ppm (δ) downfield of tetramethylsilane.

**LC/MS and HRMS analysis:** Analysis was performed on a Waters Acquity UPLC and diode array detector coupled to a Waters G2 QToF mass spectrometer fitted with a multimode ESI/APCI source. Method A: Analytical separation was carried out at 30 °C on a Phenomenex Kinetex C18 column (30 x 2.1 mm, 2.6u, 100A) using a flow rate of 0.5 mL/min in a 2 minute gradient elution with detection at 254 nm. The mobile phase was a mixture of methanol (solvent A) and water (solvent B), both containing formic acid at 0.1%. Gradient elution was as follows: 10:90 (A/B) to 90:10 (A/B) over 1.25 min, 90:10 (A/B) for 0.5 min, and then reversion back to 10:90 (A/B) over 0.15 min, finally 10:90 (A/B) for 0.1 min. Method B: Analytical separation was carried out at 30°C using a Phenomenex Kinetex C18 column (30 x 2.1 mm, 2.6u, 100A) using a flow rate of 0.3 mL/min in a 4 minute gradient elution with detection at 254 nm. The mobile phase was a mixture of methanol (solvent A) and water (solvent B), both containing formic acid at 0.1%. Gradient elution was as follows: 10:90 (A/B) to 90:10 (A/B) over 3 min, 90:10 (A/B) for 0.5 min, and then reversion back to 10:90 (A/B) over 0.3 min, finally 10:90 (A/B) for 0.2 min.

LC-HRMS method B referenced to Leucine Enkephalin fragment ion [M+H]^+^ 397.1876.

**8-Bromo-3-((2-(trimethylsilyl)ethoxy)methyl)quinazolin-4(3*H*)-one (1)**

8-Bromoquinazolin-4(3*H*)-one (304 mg, 1.35 mmol) was dissolved in anhydrous DMF (4 mL, 0.33 M). The solution was heated to 60 °C, and potassium carbonate (747 mg, 5.40 mmol) was added. After 5 min of stirring, (2-(chloromethoxy)ethyl)trimethylsilane (0.75 mL, 4.24 mmol) was slowly added and the reaction mixture stirred at 60 °C for 15 h. After cooling to room temperature the reaction mixture was diluted with EtOAc (20 mL) and washed with saturated NaHCO_3_ solution (20 mL). The aqueous layer was extracted twice more with EtOAc (2 × 20 mL) and the combined organic layers were washed with saturated LiCl solution (40 mL), saturated brine solution (40 mL), dried over MgSO_4_ and concentrated *in vacuo* to give the crude product which was purified by Biotage column chromatography on KP silica (20% EtOAc in cyclohexane) yielding product **1** as a white solid (314 mg, 65%). ^1^H NMR (500 MHz, CDCl_3_) 0.01 (s, 9H), 0.96-1.00 (m, 2H), 3.66-3.71 (m, 2H), 5.46 (s, 2H), 7.40 (t, *J* = 7.9 Hz, 1H), 8.07 (dd, *J* = 7.9, 1.4 Hz, 1H), 8.29 (s, 1H), 8.31 (dd, *J* = 7.9, 1.4 Hz, 1H); LCMS (Method A; ESI, *m/z*) *t_r_* = 1.52 min – 297, 299 [(M−SEM+TMS+H)^+^, Br isotope splitting pattern]. HRMS (Method B): found 297.0067; calculated for C_11_H_14_BrN_2_OSi (M−SEM+TMS+H)^+^ 297.0059.

**8-(4-(2-((*tert*-Butyldimethylsilyl)oxy)ethyl)-1*H*-pyrazol-1-yl)-3-((2-(trimethylsilyl)ethoxy)methyl)quinazolin-4(3*H*)-one (2)**

Bromide **1** (75 mg, 0.21 mmol), (1S,2S)-*N*1,*N*2-dimethylcyclohexane-1,2-diamine (5 mg, 0.04 mmol), copper(I) iodide (2 mg, 8.8 µmol) and anhydrous toluene (0.2 mL, 0.9 M) were added to a sealed tube containing 4-(2-((*tert*-butyldimethylsilyl)oxy)ethyl)-1*H*-pyrazole^[[1]](#footnote-1)^ (40 mg, 0.18 mmol) and potassium carbonate (51 mg, 0.37 mmol) under N_2_. The reaction mixture was stirred at 110 °C for 15h. After cooling the reaction mixture was dry-loaded onto silica for purification by Biotage column chromatography (10% EtOAc in cyclohexane) yielding the product as a clear, colourless oil (43 mg, 0.09 mmol, 48%). ^1^H NMR (500 MHz, CDCl_3_) 0.01 (s, 9H), 0.07 (s, 6H), 0.92 (s, 9H), 0.96-1.01 (m, 2H), 2.81 (t, *J* = 6.6 Hz, 2H), 3.68-3.73 (m, 2H), 3.85 (t, *J* = 6.6 Hz, 2H), 5.47 (s, 2H), 7.60 (t, *J* = 7.9 Hz, 1H), 7.66 (s, 1H), 8.19 (s, 1H), 8.25 (dd, *J* = 7.9, 1.6 Hz, 1H), 8.28 (dd, *J* = 7.9, 1.6 Hz, 1H), 8.43 (s, 1H); LCMS (Method A; ESI, *m/z*) *t_r_* = 1.88 min – 501 [(M+H)^+^]; HRMS (Method B): found 501.2714; calculated for C_25_H_41_N_4_O_3_Si_2_ (M+H)^+^ 501.2717.

**8-(4-(2-Hydroxyethyl)-1*H*-pyrazol-1-yl)-3-((2-(trimethylsilyl)ethoxy)methyl)quinazolin-4(3*H*)-one (3)**

Hydrochloric acid (4 mL, 1 M) was added to a solution of TBS-protected alcohol **2** (180 mg, 0.36 mmol) in MeOH (4 mL, 0.1 M) at 0 °C. The reaction was stirred at 0 °C for 10 min, then diluted with EtOAc (15 mL), washed with saturated NaHCO3 solution (15 mL) and saturated brine solution (15 mL), dried over MgSO4, and concentrated *in vacuo* to give the product as a white solid (112 mg, 0.29 mmol, 81%). ^1^H NMR (500 MHz, CDCl_3_) 0.01 (s, 9H), 0.95-1.00 (m, 2H), 2.85 (t, *J* = 6.4 Hz, 2H), 3.67-3.72 (m, 2H), 3.87 (t, *J* = 6.4 Hz, 2H), 5.45 (s, 2H), 7.60 (t, *J* = 7.9 Hz, 1H), 7.67 (s, 1H), 8.18 (s, 1H), 8.23 (dd, *J* = 7.9, 1.5 Hz, 1H), 8.29 (dd, *J* = 7.9, 1.5 Hz, 1H), 8.43 (s, 1H), OH signal not observed; LCMS (Method B; ESI, *m/z*) *t_r_* = 2.94 min – 387 [(M+H)^+^].

**2-(1-(4-Oxo-3-((2-(trimethylsilyl)ethoxy)methyl)-3,4-dihydroquinazolin-8-yl)-1*H*-pyrazol-4-yl)ethyl methanesulfonate (4)**

Methanesulfonic anhydride (45 mg, 0.26 mmol) was added in one portion to a solution of alcohol **3** (66 mg, 0.17 mmol) and triethylamine (0.1 mL, 0.72 mmol) in anhydrous CH2Cl2 (2.5 mL, 0.07 M) at 0 °C under N2. The reaction mixture was stirred for 15 min at 0 °C and monitored by LCMS. When the reaction was complete, the reaction mixture was quenched with saturated NaHCO3 (10 mL) solution and extracted with CH2Cl2 (3 × 10 mL). The combined organic layers were washed with saturated brine solution (30 mL), dried over MgSO4, and concentrated *in vacuo* to give mesylate **4** as a pale-yellow oil. This was used in the next step without further purification.

**8-(4-(2-(4-(3-Chlorophenyl)piperidin-1-yl)ethyl)-1*H*-pyrazol-1-yl)-3-((2-(trimethylsilyl)ethoxy)methyl)quinazolin-4(3*H*)-one (5)**

Triethylamine (0.03 mL, 0.22 mmol) was added to a solution of mesylate **4** (50 mg, 0.11 mmol, freshly made from alcohol **3**) and 4-(3-chlorophenyl)piperidine (32 mg, 0.16 mmol) in anhydrous DMF (1 mL, 0.1 M) under N2. The reaction mixture was heated at 50 °C for 15 h and monitored by LCMS. When the reaction had gone to completion, the reaction mixture was diluted in H_2_O (5 mL) and extracted three times with EtOAc (3 × 5 mL). The combined organic layers were washed with saturated LiCl solution (10 mL) and saturated brine solution (10 mL), dried over MgSO4, and concentrated *in vacuo* to give the crude material which was purified by Biotage column chromatography on KP silica (5% [0.2 M NH_3_ in MeOH] in CH_2_Cl_2_) yielding product **5** as a pale yellow oil (51 mg, 0.09 mmol, 84%). ^1^H NMR (500 MHz, CDCl_3_) 0.01 (s, 9H), 0.96-1.00 (m, 2H), 1.78-1.91 (m, 4H), 2.17 (td, *J* = 11.7, 2.5 Hz, 2H), 2.53 (tt, *J* = 11.7, 4.2 Hz, 1H), 2.67-2.74 (m, 2H), 2.80-2.88 (m, 2H), 3.17 (br d, *J* = 11.7 Hz, 2H), 3.67-3.73 (m, 2H), 5.46 (s, 2H), 7.12-7.15 (m, 1H), 7.17-7.20 (m, 1H), 7.22-7.26 (m, 2H), 7.60 (t, *J* = 7.9 Hz, 1H), 7.67 (s, 1H), 8.20 (s, 1H), 8.25 (dd, *J* = 7.9, 1.5 Hz, 1H), 8.29 (dd, *J* = 7.9, 1.5 Hz, 1H), 8.42 (s, 1H); LCMS (Method A; ESI, *m/z*) *t_r_* = 1.27 min – 564, 566 [(M+H)^+^, Cl isotope splitting pattern]; HRMS (Method B): found 564.2576; calculated for C_30_H_39_ClN_5_O_2_Si (M+H)^+^ 564.2562.

**8-(4-(2-(4-(3-Chlorophenyl)piperidin-1-yl)ethyl)-1*H*-pyrazol-1-yl)quinazolin-4(3*H*)-one (CCT366293)**

Hydrochloric acid (6 M, 1 mL) was added to a solution of protected quinazoline **5** (44 mg, 0.08 mmol) in THF (1 mL, 0.1 M). The reaction mixture was stirred at 50 °C for 3 h and monitored by LCMS. Following completion of the reaction, the reaction mixture was concentrated *in vacuo* and purified by Biotage column chromatography on a KP-NH snap column eluting with 0-40% EtOH in CH_2_Cl_2_. Following column chromatography the material was triturated with Et_2_O to give **CCT366293** as a white solid (27 mg, 0.06 mmol, 81%). ^1^H NMR (500 MHz, DMSO-*d*_6_) 1.66 (qt, *J* = 12.2, 3.4 Hz, 2H), 1.74-1.80 (m, 2H), 2.07 (td, *J* = 11.6, 2.2 Hz, 2H), 2.52-2.60 (m, 3H), 2.67-2.73 (m, 2H), 3.06 (br d, *J* = 11.6 Hz, 2H), 7.22-7.26 (m, 2H), 7.30-7.35 (m, 2H), 7.61 (t, *J* = 8.0 Hz, 1H), 7.68 (s, 1H), 8.10 (dd, *J* = 8.0, 1.5 Hz, 1H), 8.16 (dd, *J* = 8.0, 1.5 Hz, 1H), 8.21 (s, 1H), 8.53 (s, 1H), 12.47 (br s, 1H); LCMS (Method A; ESI, *m/z*) *t_r_* = 1.27 min – 434 [(M+H)^+^-for ^35^Cl]; HRMS (Method B): found 434.1741; calculated for C_24_H_25_ClN_5_O (M+H)^+^ 434.1747.

1. Prepared according to Bavetsias, V. *et al*. *J Med Chem* **2016**, *59*, 1388-1409. [↑](#footnote-ref-1)
